# Proficiency of Clear Aligner Therapy: A Systematic Review and Meta-Analysis

**DOI:** 10.7759/cureus.45072

**Published:** 2023-09-11

**Authors:** Akshay Shrivastava, Pritam Mohanty, Bhagabati P Dash, Sanghamitra Jena, Nivedita Sahoo

**Affiliations:** 1 Department of Orthodontics and Dentofacial Orthopaedics, Kalinga Institute of Dental Sciences, Kalinga Institute of Industrial Technology (KIIT) (Deemed to be University), Bhubaneswar, IND; 2 Department of Orthodontics, Kalinga Institute of Dental Sciences, Kalinga Institute of Industrial Technology (KIIT) (Deemed to be University), Bhubaneswar, IND

**Keywords:** orthodontic treatment dental esthetics, digital dentistry, digital planning, outcome, treatment, orthodontic therapy, orthodontics, invisalign, clear aligners, malocclusion

## Abstract

The aim of this study was to evaluate the efficacy and efficiency of orthodontic treatment using clear aligner therapy (CAT). This efficiency was measured using the Peer Assessment Rating (PAR) index, the American Board of Orthodontics (ABO) index, or the similarity between the final ClinCheck and the final scanned models. A search was done electronically between 1998 and 2021 using the Cochrane Library, PubMed, and Google Scholar databases. Three reviewers individually rated the articles. The ROBINS tool and the Cochrane risk of bias tool were used to evaluate the quality of observational research and randomized controlled trials, respectively. The degree of certainty for each selected outcome was evaluated using the grading of recommendations assessment, development, and evaluation (GRADE) approach.* *Six studies with a total of 166 participants were considered after the full texts of 61 potential reports were reviewed. This research included in the review covered the period from 1998 to 2021 in retrospect. According to the current systematic review and meta-analysis, transparent aligners had a successful outcome. In mild to moderate cases, aligner treatment appears to have a significant advantage in terms of efficiency (treatment time); nonetheless, insufficient evidence of efficacy was observed based on multiple cross-sectional investigations. When compared to traditional brackets, clear aligners provided a more stable course of treatment.

## Introduction and background

Although their underlying concept dates back to the early 20th century, transparent aligners are not a particularly novel orthodontic treatment option. Starting with Remensnyder's [1] "Flex-O-Tite" appliance, Kesling [2] developed the idea of employing tooth positioners in a sequential manner for incremental tooth movements. The first transparent thermoplastic appliance for orthodontic tooth movement was created by Nahoum [3] in the 1960s. Based on his idea, Ponitz produced the first "invisible retainer" [4]; in the 1980s, McNamara made improvements. The Essix retainer, which Sheridan invented in 1993, is a similar device [5]. Since then, with the development of the digital era, we have been able to integrate state-of-the-art technology with these earlier theoretical foundations to offer a selection of transparent aligner systems that enable a more comprehensive approach to orthodontic treatment [6,7].

Material and methodology

In compliance with PRISMA Guidelines 2020, the study was carried out using the checklist below.

Eligibility Criteria

The study's inclusion criteria called for:

• Systemically and mentally healthy patients with permanent dentition (minimum age of 15 years), who had received clear aligner orthodontic treatment.

· Clear aligners were used to treat the patient.

Exclusion criteria contain studies including:

• Patients with mixed or deciduous teeth.

• Those who have not received clear aligner orthodontic therapy.

• Those with systemic illnesses that raise their risk of infection and affect their findings.

Participants/Population

Participants had to meet the minimum chronological age requirement of 15 years and be in overall and mental good health. They are also required to have received clear aligner orthodontic treatment.

Exclusion criteria for research included:

• Patients with mixed or deciduous teeth.

• People who had not received clear aligner orthodontic treatment. 

• Patients with systemic disorders, which increase the risk of infections and have negative effects on outcomes.

• Patients receiving clear aligners treatment.

Comparator(s)/Control

Self-evaluation was conducted both before and after clear aligner therapy (CAT), which serves as the baseline.

Types of Study Included

Included papers: Records of orthodontic treatment with clear aligners for dental malocclusion published in the English language, prospective cohort studies, retrospective studies, and randomized and non-randomized clinical trial studies.

Omitted studies: Reviews, case series studies, reviews, case reports (in-vitro), (cadaveric and animal studies), and others were not included.

Outcome

The effectiveness and efficacy of orthodontic treatment carried out using CAT were the main topics of this systematic study. This efficiency was assessed using the Peer Assessment Rating (PAR) index, the American Board of Orthodontics (ABO) index, or the equivalence between the Final ClinCheck and the final scanned models.

## Review

Priority Reporting Items of the Extended Meta-Analysis for Systematic Reviews and Diagnostic Test Accuracy (PRISMA-DTA) guidelines were performed as per the protocol for any systematic review and are registered in the National Institutes of Health database (www.crd.york.ac.uk/Prospero, logs: CRD42023378828).

PICO of the study

P: Patients with malocclusion

I: Clear aligners

C: Conventional methods

O: Tooth movement

Data were extracted from PubMed, Scopus, Embase, Medline, and Publons. Two reviewers conducted the process of screening and selecting studies independently. The initial selection of studies for inclusion was made based on titles and abstracts, and potentially eligible studies were identified. The data extracted were entered into Excel spreadsheets, and duplicates were removed. Divergences of opinion between the reviewers were resolved through discussion with a third reviewer.

Literature search strategy

PubMed, Medline, and Scopus were the electronic databases used for the search. In order to search for publications on PubMed and Medline, the boolean operators "AND" and "OR" were utilized using phrases such as "malocclusion," "dental malocclusion," "orthodontic patients," "angle," "classification," "cross bite," and "tooth crowding." The usage of CAT, thermoplastic orthodontics, clear aligner appliances, clear dental braces, and Invisalign were all employed (Table 1). The boolean operators "OR" and "AND" were utilized to combine the mesh phrases for malocclusion and clear aligners that were found in PubMed. Boolean operators are simple words that are combined with other words to combine or omit terms from a search, resulting in more specialized and helpful results. Additional proximity operators were deployed. Education Pre/2 literacy is the prefix. For Scopus, loose phrases with double were used, instead of separate words, e.g., “Effectiveness of clear aligners?” and “Efficacy of clear aligners?.”

**Table 1:** The search strategy on the basis of the PICO format

Participant	Intervention	Comparison	Outcome
Malocclusion, dental malocclusion patients undergoing orthodontic treatment, angle's classification, cross bite, crossbite, tooth crowding	Clear aligner therapy, orthodontic treatment by a thermoplastic material, clear aligner appliances, clear dental braces, Invisalign	Self-comparison	Effectiveness of clear aligners, efficacy of clear aligners

Table 2 depicts the keywords used for data extraction.

**Table 2:** Medical subject heading (MeSH) terms for malocclusion and Invisalign

	Entry Terms
MeSH terms for malocclusion tree number(s): C07.793.494 MeSH unique ID: D008310	Malocclusions, crowding of the teeth, crowding of the teeth, crossbites of the teeth, and crossbites of the teeth, Classification of Angles, Classification of Angles, Classification of Angles
MeSH term for Invisalign tree number(s): E06.658.453.578 MeSH unique ID: D009968	Appliances, Removable Orthodontic Appliance, Removable Orthodontic Appliances, Orthodontic Appliance, Removable Appliances, Appliances, Appliances, Appliances, Appliances, Appliances, Appliances, Appliances, Clear AlignerClear Aligner Appliance, Clear Dental Braces, Brace, Clear Dental, Braces, Invisalign, Clear Dental Braces, Clear Dental Braces, and Dental Braces

The data extraction strategy is shown below in Table 3.

**Table 3:** Data extraction strategy

Database	No. of articles	Date
Pubmed	323	01/12/2022
Medline	0	02/12/2022
Web of Science	3	03/12/2022
Scopus	5	03/12/2022

Result

An additional examiner cross-checked the 331 articles that were acquired via the electronic search during the years 1998-2021 to eliminate any duplicates. After a double inspection, eight pieces were eliminated. After 323 papers were checked for title and abstract, 201 were eliminated, and 122 were then looked at in light of the research question. For the qualitative analysis, nine papers that complied with the review's PICO format were included.

**Figure 1:**
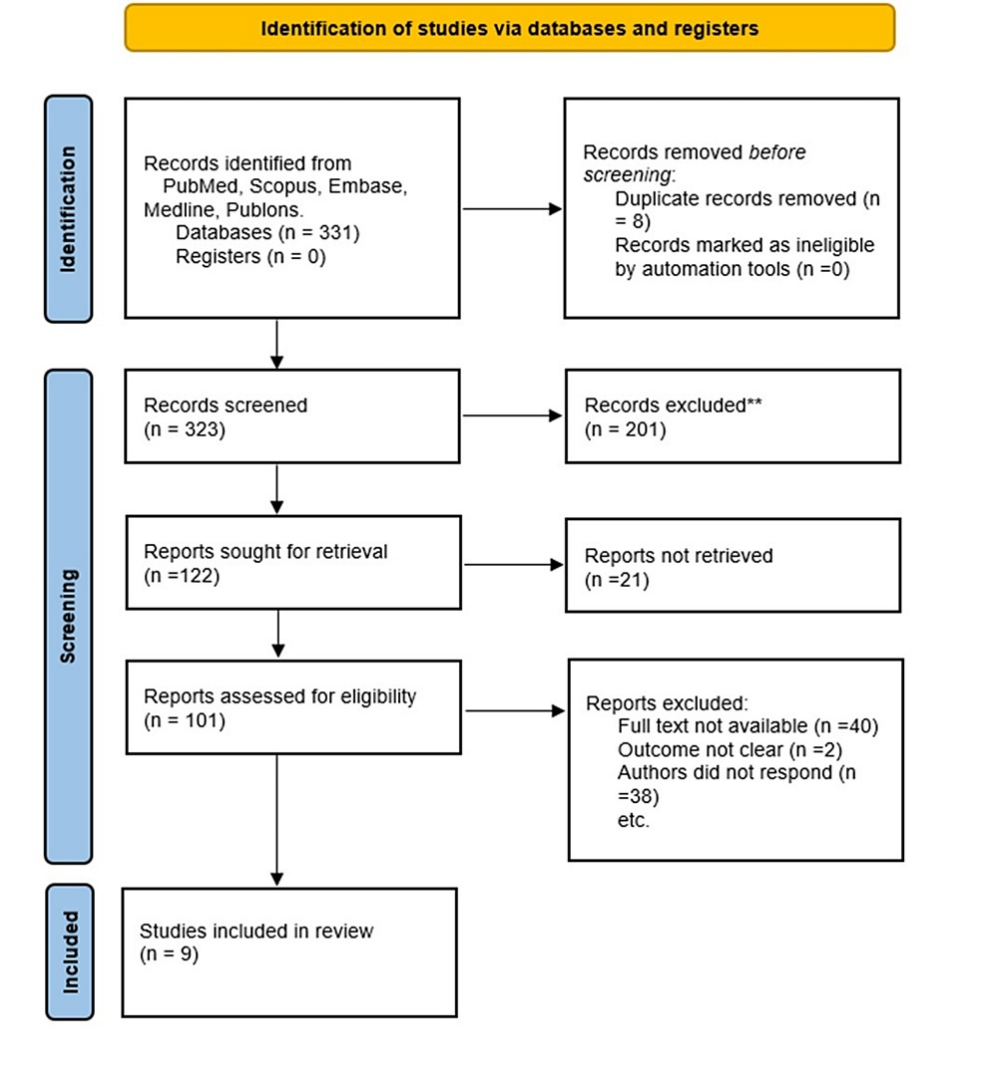
PRISMA flow diagram

The identification of studies via databases and registers has been shown in the following (Figure 1).

Included studies: After reviewing the whole texts of 61 possible reports, six studies with a combined total of 166 individuals were taken into consideration. For the review, studies spanning 1998 to 2021 were retrospectively examined.

Risk of bias: The ROBIN technique was used to calculate the bias risk in interventions and non-randomized studies. In all the studies that were included in the report, the risk of bias was generally minimal.

Meta-Analysis

Meta-analysis was calculated for the objective grading system (OGS). Two studies were included, which were homogenous and were not significant. Meta-analysis was used on time point, which was heterogeneous.

Discussion

In previous studies, 26 limited the indications for treatment with clear aligners to cases of simple, mild malocclusion, but advances in factors critical to the quality and function of many aligners have led to the possibility of using aids such as divots and other means of movement, their different properties, and the possibilities of different types of tooth movements [8-13].

The availability of 3D scanners, 3D printers, and a variety of validated 3D printing materials in dental offices and with orthodontic treatment planning software has enabled clinicians to fabricate their patients' aligners directly, or in their clinics, without having to collaborate with CA providers [14-20]. In a study by Rossini et al. [21], notably, the least amount of movement occurs when the upper and lower anterior teeth need to be extruded. Additionally, it was discovered that the rotation of the upper incisors and canines was inaccurate [22]. Sfondrini et al.'s study [23], which conducted the above analysis, found that, although movement was good overall, rotation was the least common movement, especially in the upper incisors, canines, premolars, and molars. Last but not least, a "bite ramp" was positioned just on the buccal side in cases of crossbite in order to correct overbite on the lingual surface of the upper teeth [24-29]. Additional materials (such as rubber bands or microscrews) can be added to the body, making the CAT more versatile and flexible. Fifty-seven orthodontic nubs were cut into the aligner or transferred to the aligner itself (can be used as direct anchors for elastics). Intermaxillary elastic materials are commonly used in class II orthodontic malocclusions and subdental orthodontics for good strength control and reasonable oral control. Orthodontic mini-screws provide unilateral distalization of class II malocclusions with a single bone-loading object during hybrid therapy, enabling treatment with clear aligners comparable to that used for class III malocclusions in the following stages [30]. Other studies [31-33] reported pain more than a day after initial archwire placement, and some studies reported greater pain in the product treatment group [34], but within 24 hours of being stable. Control and Invisalign treatment groups experienced greater and less pain for similar durations, although one study found greater pain in the 77-plaque group.

Joe et al. reported that 44 patients (mean age: 26.4 ± 7.7 years) were randomly assigned to either the fixed labial appliance or the clear aligner group in a 1:1 ratio. Both groups have similar baseline characteristics: Fixed appliance mean crowding was 2.1 ± 1.3 mm versus clear aligner mean crowding of 2.5 ± 1.3 mm; fixed appliance mean mandibular incisor inclination was 90.8 ± 5.4 u vs 91.6 ± 6.4u for the clear aligner group. Mandibular incisor proclination was 5.3 ± 4.3u with fixed appliances. The mandibular incisors were proclined by 3.4 ± 3.2u using clear aligners. There was no statistically significant difference between the two groups (p>0.05). The study concluded that there was no difference in the amount of mandibular incisor proclination induced by transparent aligners versus fixed labial appliances in mild cases [34].

The study conducted by Orfeas et al. evaluated the accuracy of various tooth movements during orthodontic treatment. They observed that the horizontal shifts of all incisors were precise, showing minimal differences (ranging from 0.20 mm to 0.25 mm) between the anticipated and actual movements. However, vertical adjustments, especially the downward repositioning (intrusion) of upper central incisors, exhibited lower accuracy. The median variance between predicted and achieved intrusion was 1.5 mm, indicating inconsistencies between the two. Additionally, the study noted that accomplished tooth rotations were notably smaller than predicted rotations. Notably, the maxillary canines displayed the most significant difference, with a 3.05-degree gap between anticipated and achieved rotation [35].

Limitations

Since there are so few RCTs and a large research gap between the studies, more research could be further conducted.

## Conclusions

It is evident that the field of orthodontics is also developing with technological innovation, with methods such as 3D printing, teleorthodontics, and biopolymers, leading to the changes made to the orthodontic landscape. However, it is impossible to adopt all the changes that are occurring at once; caution and restraint are the most crucial guidelines that must be followed. On the basis of multiple cross-sectional studies, the results of the systematic review demonstrated that CAT was a more successful way of treatment for mild to moderate instances. The aforementioned conclusion will need to be supplemented by additional studies due to the constantly evolving methodologies used in CAT.

## References

[1] Remensnyder O (1926). A gum-massaging appliance in the treatment of pyorrhea. Dent Cosmos.

[2] Kesling HD (1945). The philosophy of the tooth positioning appliance. Am J Orthod Dentofacial Orthop.

[3] Nahoum HI (1964). The vacuum formed dental contour appliance. NY State Dent J.

[4] Ponitz RJ (1971). Invisible retainers. Am J Orthod.

[5] Sheridan JJ (1993). Essix retainers: fabrication and supervision for permanent retention. J Clin Orthod.

[6] Simon M, Keilig L, Schwarze J, Jung BA, Bourauel C (2014). Forces and moments generated by removable thermoplastic aligners: incisor torque, premolar derotation, and molar distalization. Am J Orthod Dentofacial Orthop.

[7] Barone S, Paoli A, Razionale AV, Savignano R (2017). Computational design and engineering of polymeric orthodontic aligners. Int J Numer Method Biomed Eng.

[8] Djeu G, Shelton C, Maganzini A (2005). Outcome assessment of Invisalign and traditional orthodontic treatment compared with the American Board of Orthodontics objective grading system. Am J Orthod Dentofacial Orthop.

[9] Buschang PH, Shaw SG, Ross M, Crosby D, Campbell PM (2014). Comparative time efficiency of aligner therapy and conventional edgewise braces. Angle Orthod.

[10] Baldwin DK, King G, Ramsay DS, Huang G, Bollen AM (2008). Activation time and material stiffness of sequential removable orthodontic appliances. Part 3: premolar extraction patients. Am J Orthod Dentofacial Orthop.

[11] Melkos AB (2005). Advances in digital technology and orthodontics: a reference to the Invisalign method. Med Sci Monit.

[12] Tepedino M, Paoloni V, Cozza P, Chimenti C (2018). Movement of anterior teeth using clear aligners: a three-dimensional, retrospective evaluation. Prog Orthod.

[13] White DW, Julien KC, Jacob H, Campbell PM, Buschang PH (2017). Discomfort associated with Invisalign and traditional brackets: a randomized, prospective trial. Angle Orthod.

[14] Damasceno Melo PE, Bocato JR, de Castro Ferreira Conti AC, Siqueira de Souza KR, Freire Fernandes TM, de Almeida MR, Pedron Oltramari PV (2021). Effects of orthodontic treatment with aligners and fixed appliances on speech: a randomized clinical trial. Angle Orthod.

[15] Albhaisi Z, Al-Khateeb SN, Abu Alhaija ES (2020). Enamel demineralization during clear aligner orthodontic treatment compared with fixed appliance therapy, evaluated with quantitative light-induced fluorescence: a randomized clinical trial. Am J Orthod Dentofacial Orthop.

[16] Nahoum HI (2014). Forces and moments generated by removable thermoplastic aligners. Am J Orthod Dentofacial Orthop.

[17] Levrini L, Mangano A, Montanari P, Margherini S, Caprioglio A, Abbate GM (2015). Periodontal health status in patients treated with the Invisalign(®) system and fixed orthodontic appliances: a 3 months clinical and microbiological evaluation. Eur J Dent.

[18] Chhibber A, Agarwal S, Yadav S, Kuo CL, Upadhyay M (2018). Which orthodontic appliance is best for oral hygiene? A randomized clinical trial. Am J Orthod Dentofacial Orthop.

[19] Dai FF, Xu TM, Shu G (2019). Comparison of achieved and predicted tooth movement of maxillary first molars and central incisors: first premolar extraction treatment with Invisalign. Angle Orthod.

[20] Ke Y, Zhu Y, Zhu M (2019). A comparison of treatment effectiveness between clear aligner and fixed appliance therapies. BMC Oral Health.

[21] Rossini G, Parrini S, Castroflorio T, Deregibus A, Debernardi CL (2015). Efficacy of clear aligners in controlling orthodontic tooth movement: a systematic review. Angle Orthod.

[22] Zinelis S, Panayi N, Polychronis G, Papageorgiou SN, Eliades T (2022). Comparative analysis of mechanical properties of orthodontic aligners produced by different contemporary 3D printers. Orthod Craniofac Res.

[23] Sfondrini MF, Gandini P, Castroflorio T (2018). Buccolingual inclination control of upper central incisors of aligners: a comparison with conventional and self-ligating brackets. Biomed Res Int.

[24] Flores-Mir C, Brandelli J, Pacheco-Pereira C (2018). Patient satisfaction and quality of life status after 2 treatment modalities: Invisalign and conventional fixed appliances. Am J Orthod Dentofacial Orthop.

[25] Weir T (2017). Clear aligners in orthodontic treatment. Aust Dent J.

[26] Tartaglia GM, Mapelli A, Maspero C, Santaniello T, Serafin M, Farronato M, Caprioglio A (2021). Direct 3D printing of clear orthodontic aligners: current state and future possibilities. Materials (Basel).

[27] Jindal P, Worcester F, Siena FL, Forbes C, Juneja M, Breedon P (2020). Mechanical behaviour of 3D printed vs thermoformed clear dental aligner materials under non-linear compressive loading using FEM. J Mech Behav Biomed Mater.

[28] Levrini L, Novara F, Margherini S, Tenconi C, Raspanti M (2015). Scanning electron microscopy analysis of the growth of dental plaque on the surfaces of removable orthodontic aligners after the use of different cleaning methods. Clin Cosmet Investig Dent.

[29] (2023). Vacuum molding machine. https://scholar.google.com/scholar?q=intitle:Lawrence%20BR%2C%20inventor%3B%20AUTO%20VAC%20Co%2C%20assignee.%20Vacuum%20molding%20machine.%20United%20States%20patent%20US%202%2C814%2C074.

[30] Kuncio D, Maganzini A, Shelton C, Freeman K (2007). Invisalign and traditional orthodontic treatment postretention outcomes compared using the American Board of Orthodontics objective grading system. Angle Orthod.

[31] Patterson BD, Foley PF, Ueno H, Mason SA, Schneider PP, Kim KB (2021). Class II malocclusion correction with Invisalign: is it possible?. Am J Orthod Dentofacial Orthop.

[32] Jones M, Chan C (1992). The pain and discomfort experienced during orthodontic treatment: a randomized controlled clinical trial of two initial aligning arch wires. Am J Orthod Dentofacial Orthop.

[33] Jones ML (1984). An investigation into the initial discomfort caused by placement of an archwire. Eur J Orthod.

[34] Hennessy J, Garvey T, Al-Awadhi EA (2016). A randomized clinical trial comparing mandibular incisor proclination produced by fixed labial appliances and clear aligners. Angle Orthod.

[35] Charalampakis O, Iliadi A, Ueno H, Oliver DR, Kim KB (2018). Accuracy of clear aligners: a retrospective study of patients who needed refinement. Am J Orthod Dentofacial Orthop.

